# Irinotecan Plus Doxorubicin Hydrochloride Liposomes for Relapsed or Refractory Wilms Tumor

**DOI:** 10.3389/fonc.2021.721564

**Published:** 2021-09-21

**Authors:** Juan Wang, Lian Zhang, Lanying Guo, Yi Que, Yu Zhang, Feifei Sun, Jia Zhu, Suying Lu, Junting Huang, Liuhong Wu, Ruiqing Cai, Zijun Zhen, Sihui Zeng, Yizhuo Zhang, Xiaofei Sun

**Affiliations:** ^1^Department of Pediatric Oncology, Sun Yat-sen University Cancer Center, Guangzhou, China; ^2^Department of Pediatric Oncology, The Fifth Affiliated Hospital of Guangzhou Medical Guangzhou, Guangzhou, China; ^3^Department of Pathology, Sun Yat-sen University Cancer Center, Guangzhou, China; ^4^Department of Medical Imaging, Sun Yat-sen University Cancer Center, Guangzhou, China

**Keywords:** Wilms tumor, pediatric tumor, salvage regimen, irinotecan, doxorubicin hydrocloride liposome

## Abstract

**Purpose:**

The prognosis of relapsed or refractory pediatric Wilms tumor (WT) is dismal, and new salvage therapies are needed. This study aimed to evaluate the efficacy of the combination of irinotecan and a doxorubicin hydrochloride liposome regimen for relapsed or refractory pediatric WT.

**Patients and Methods:**

The present study enrolled relapsed or refractory pediatric WT patients who were treated with the AI regimen (doxorubicin hydrochloride liposomes 40 mg/m^2^ per day, day 1, and irinotecan 50 mg/m^2^ per day with 90-min infusion, days 1–5; this regimen was repeated every 3 weeks) at Sun Yat-sen University Cancer Center from July 2018 to September 2020. The response was defined as the best-observed response after at least two cycles according to the Response Evaluation Criteria of Solid Tumors (RECIST 1.1), and toxicity was evaluated according to the Common Terminology Criteria for Adverse Events (CTCAE 4.03).

**Results:**

A total of 16 patients (male:female, 8:8) with a median age of 4.2 years (0.5–11 years) with relapsed or refractory disease were enrolled in this study, including 14 patients with relapsed disease and two patients with refractory disease. These patients received 1–8 courses (median, 3 courses) of the AI regimen. Fourteen patients were assessable for response: two with complete response (CR), five with partial response (PR), two with stable disease (SD), and five with progressive disease (PD). The objective response rate was 50% (two CR, five PR), and the disease control rate was 64% (two CR, five PR, and two SD). Seven out of 14 patients (50%) were alive at the last follow-up, ranging from 2.6 to 32.4 months. The median progression-free survival and median overall survival were 3.5 months (range 0.5–12 months) and 8 months (range 1–28 months), respectively. Sixteen patients were assessable for toxicity, with the most common grade 3 or 4 adverse events being alopecia (62%), leukopenia (40%), abdominal pain (38%), diarrhea (23%), and mucositis (16%), etc. No fatal adverse events have been observed, and modest adverse effects can be administered.

**Conclusion:**

Irinotecan and doxorubicin hydrochloride liposome regimens have positive efficacy on relapsed or refractory pediatric WT with well-tolerated toxicity. A prospective clinical trial is warranted.

## Introduction

Wilms tumor (WT) is an embryonal tumor that accounts for 90% of childhood renal tumors (1, 2). Medical advances have greatly improved the survival rate of children diagnosed with WT in recent decades, such as the AREN0533 study, which showed that excellent overall survival (OS) was achieved after omission of primary lung radiotherapy (RT) in patients with lung nodule complete response, and event-free survival (EFS) was significantly improved in patients with lung nodule incomplete response using four cycles of cyclophosphamide/etoposide in addition to vincristine/actinomycin D/doxorubicin (DD4A) drugs. The survival rate of advanced-stage WT treated according to the Children’s Oncology Group (COG) AREN0533 protocol was over 90%, with the overall relapse rate decreasing to less than 15% (3). However, the prognosis of relapsed or refractory patients is still dismal. Conventional surgery, RT, and chemotherapy, such as the combination of actinomycin D and vincristine and/or doxorubicin, are generally used as standard therapies for WT (4–6). Relapsed WT is clinically heterogeneous, and the prognosis of patients treated with standard-dose chemotherapy and radiation was better than that of patients with adverse prognostic features, including unfavorable histology, relapse less than 12 months from diagnosis, and initial treatment with three-drug chemotherapy (4, 5). Studies have shown that high-dose chemotherapy (HDT) followed by autologous hematopoietic stem cell rescue (HSCR) can improve the prognosis of relapsed WT patients (7, 8). However, relapsed WT patients rarely receive HDT followed by HSCR in China. Few effective chemotherapeutic agents are available for relapsed and refractory patients who have previously received multidrug chemotherapy (such as ifosfamide, carboplatin, etoposide, cyclophosphamide, doxorubicin), and new salvage chemotherapy agents need to be explored. Limited options are available for these types of patients due to toxicity and side effects on bone marrow, cardiac function, and impaired function of the liver and kidney (9–11).

Irinotecan, a topoisomerase I inhibitor, is a semisynthetic analog of camptothecin with modest toxicity appearing as myelosuppression, controllable non-hematologic side effects, and powerful effectivity against pediatric solid tumors in both a xenograft model and patients (12–15). Irinotecan combined with other chemotherapy agents (such as vincristine, temozolomide, bevacizumab) has been reported in clinical applications to treat pediatric solid cancer, including a subset of patients with relapsed WT (16–21). The results of the COG AREN0321 Study showed that the overall response rate (ORR) for the VI regimen (irinotecan combined with vincristine) used to treat newly diagnosed diffuse anaplastic Wilms tumor (DAWT) was 79% (22). For relapsed or refractory nephroblastoma, several retrospective clinical studies have shown that irinotecan-containing regimens have positive clinical efficacy and tolerable toxicity (21, 23–25). Doxorubicin hydrochloride liposomes are a novel formulation of doxorubicin encapsulated in polyethylene glycol-coated liposomes and were designed to enhance the efficacy and reduce the dose-limiting toxicities of conventional doxorubicin (26). Research has shown that the ORR of doxorubicin hydrochloride liposomes alone for pediatric sarcoma is 37.5% (27). We attempted to treat relapsed or refractory WT with an irinotecan–doxorubicin hydrochloride liposome (AI) regimen in July 2018. In this study, we retrospectively analyzed the efficacy and toxicity of the AI regimen in 16 patients with relapsed or refractory WT from July 2018 to September 2020.

## Materials And Methods

### Patients

From July 2018 to September 2020, 16 pediatric patients with relapsed and refractory WTs who received a doxorubicin hydrochloride liposome plus irinotecan regimen at Sun Yat-sen University Cancer Center were selected and included in the analysis. The inclusion criteria were as follows: (1) patients with relapsed and refractory WT aged ≤18 years; (2) doxorubicin hydrochloride liposome plus irinotecan chemotherapy regimen; and (3) complete clinical data. The exclusion criteria were patients who had previously received chemotherapy with doxorubicin-containing liposomes or irinotecan regimens or patients with grade ≥2 cardiac function insufficiency.

This study was approved by the Sun Yat-sen University Cancer Center Ethical Review Board (B2021-071-01).

### Treatment Schedule

The frontline treatment of WT was performed according to the National Wilms Tumor Study (NWTS)-5 protocol. According to the NWTS-5 protocol, stage I/II WT with favorable histology was treated with the actinomycin D and vincristine (VA) regimen for frontline treatment; cyclophosphamide, pirarubicin, and vincristine (CAV) alternating with carboplatin and etoposide (CE) for the first relapse; and VIP (ifosfamide, cisplatin, and etoposide) regimen or AI regimen for the second relapse. Stage III/IV WT with favorable histology was treated with the actinomycin D, pirarubicin, and vincristine (VAD) regimen for initial chemotherapy; CAV alternating with CE for the first relapse; and VIP regimen or AI regimen for the second relapse. If the lung metastases were PD after frontline chemotherapy, we delayed the whole lung RT until the lesions shrunk or were removed for stage IV patients. For relapsed patients, surgery or RT was given if necessary. Patients with relapsed or refractory WT received an AI regimen until disease progression, unacceptable toxicity, or patient withdrawal, but no more than eight courses, and were evaluated for efficacy every two cycles. The AI regimen included doxorubicin hydrochloride liposomes (40 mg/m^2^ per day with more than 30-min infusion, day 1) and irinotecan (50 mg/m^2^ per day with 90-min infusion, days 1–5), repeated every 3 weeks. Doxorubicin hydrochloride liposomes should be given with anti-allergic pretreatment (including cimetidine, dexamethasone, diphenhydramine, or Benadryl) half an hour prior; atropine was routinely administered 30 min before irinotecan. Before the AI regimen started, standard chemotherapy consent was signed by the guardian of all patients. Surgery was performed during or after the AI regimen, according to the response to the AI regimen and the opinion of the surgeon, and RT was performed after the AI regimen or surgery if necessary.

### Stage and Pathology

The clinical stage was based on the COG staging system. Initial pathology was performed according to the COG protocol and classified into favorable histology (FH) group and an unfavorable histology (UFH) group. The FH group was classified into four subtypes: mesenchymal, epithelial, blastemal predominant, and mixed. The UFH group included diffuse anaplasia and focal anaplasia.

### Efficacy and Toxicity Evaluation

The response was defined as the best-observed response after at least two cycles of the doxorubicin hydrochloride liposome plus irinotecan regimen. The efficacy evaluation of all patients was evaluated after the AI regimen and before surgery and RT. A CT scan was used to evaluate the recurrent lesions in the thoracic region or abdomen, and MRI was used for the recurrent lesions in the bone. According to the Response Evaluation Criteria of Solid Tumors (RECIST) standard for efficacy evaluation, analysis was divided into complete response (CR), partial response (PR), stable disease (SD), and progressive disease (PD). Progression-free survival (PFS) was defined as the time from the start of the doxorubicin hydrochloride liposome plus irinotecan regimen to the progression of the disease or the time of the last follow-up. OS was defined as the time from the start of the doxorubicin hydrochloride liposome plus irinotecan regimen to death or the last follow-up.

Toxicity assessment is based on the Common Terminology Criteria for Adverse Events (CTCAE 4.03).

### Statistical Analysis

SPSS software version 22.0 (IBM, Chicago, IL) was used for statistical analysis, and the Kaplan–Meier method was used to calculate the OS rate and PFS rate.

## Results

### Patient Characteristics

A total of 16 patients (male:female, 8:8) diagnosed with relapsed or refractory WT were enrolled in this study, including 14 patients with relapsed disease and two patients with refractory disease, with a median age of 4.2 years (0.5–11 years) at relapsed or refractory disease and a median time of 17.5 months (7–108 months) between tumor diagnosis and relapsed or refractory disease. Most of the patients had advanced-stage disease at initial diagnosis (stage II: N = 4, stage III: N = 6, stage IV: N = 5), and one patient had bilateral disease of the kidney at initial diagnosis. The histology of all the patients at initial diagnosis was classified as the FH group. All the patients had received multiple salvage regimens before receiving AI regimen chemotherapy. The cumulative doses of doxorubicin were 150–400 mg/m^2^ (median 250 mg/m^2^) (**Table 1**). There were no differences in the cumulative amounts of anthracycline between the four stage II patients at diagnosis and the other patients regarding cumulative doxorubicin doses before the start of AI regimen, because after the recurrence of the four stage II patients, DOXO-containing salvage chemotherapy regimen was used before the AI regimen. These patients received 1–8 courses (median, three courses) of the AI regimen.

**Table 1:** Clinical characteristics of the recruited pediatric patients with relapsed/refractory Wilms tumor (n = 16).

Characteristics	No. of patients (%)
Sex	
Male	8 (50%)
Female	8 (50%)
Age (years)	
Median	4.2
Range	0.5–11
Disease status at start of AI regimen	
Relapse	4 (87.5%)
Refractory	2 (12.5%)
No. of chemotherapy lines	
1	0
2	10 (62.5%)
≥3	6 (37.5%)
No. of courses per patient	
Median	3
Range	1–8
Accumulative doses of pirarubicin (THP) mg/m^2^	
Median	250
Range	150–400

Before the start of the AI regimen, isolated local recurrence occurred in two patients, isolated lung metastasis occurred in nine patients, and both local and distant metastases (including liver, lung, pelvic cavity, and bone) occurred in five patients.

### Response and Survival

The duration from the initial treatment with the AI regimen to subsequent PD was 0.5–12 months (median, 3.5 months). Two patients were not evaluable for response, including patient #15 who was lost to follow-up after only one course of the AI regimen and patient #16 with no evaluable lesions. With a median of three cycles of the AI regimen (1–8 cycles), 14 patients were assessable for response: two CR, five PR, two SD, and five PD (**Table 2**).

**Table 2:** The response to AI regimen.

Disease status at the start of regimen	No. of patients	Response (No.)			
		CR	PR	SD	PD
Refractory disease	1	0	1	0	0
Relapse	13	2	4	2	5
Total	14	2 (14.3%)	5 (35.7%)	2 (14.3%)	5 (35.7%)

AI, doxorubicin hydrochloride liposome, irinotecan; CR, complete response; PD, progressive disease; PR, partial response; SD, stable disease.

With a median follow-up time of 10.5 months (1.1–34.8 months), seven out of 14 patients (50%) were alive at the last follow-up, ranging from 2.6 to 32.4 months. Both patients who achieved CR were alive at the last follow-up (patient #6, who reached CR after six courses of the AI regimen, showed tumor relapse after 5 months, then received other salvage chemotherapies, including topotecan and cyclophosphamide, and was alive with the disease for 28.8 months at the last follow-up; patient #12 achieved CR after eight cycles of the AI regimen and was alive at the last follow-up, showing no evidence of disease for 9.2 months).

Of the five patients who achieved PR (after 4–6 courses of the AI regimen), four patients achieved CR after further clinical management (patients #3 and #5 received surgery and irradiation of pulmonary lesions, patients #13 and #14 received whole-lung irradiation), and one patient (patient #8) achieved PR after four courses of the AI regimen but PD after six courses and then received other salvage therapies. Of the five patients who achieved PR after the AI regimen, three patients (patients #3, #5, and #14) were alive without disease, one patient (patient #8) was alive with disease, and one (patient #13) patient died of disease at the last follow-up.

Both patients (patients #2 and #4) who achieved SD (after 2–3 courses of the AI regimen) changed to a further salvage chemotherapy regimen but died of tumor progression at the last follow-up.

Of the five patients who achieved PD, patient #7 underwent removal of the lung lesions and then received radiation of the whole lung and was alive at the last follow-up for 3.7 months. The other four patients (patients #1, #9, #10, and #11) died of disease progression at the last follow-up. Patient #13 achieved PR after receiving six cycles of AI regimen, but the patient refused surgery because the whole lung needed to be removed to achieve complete removal of the tumor. Then, the patient received whole-lung RT but relapsed after 7 months and died of disease.

**Figure 1:**
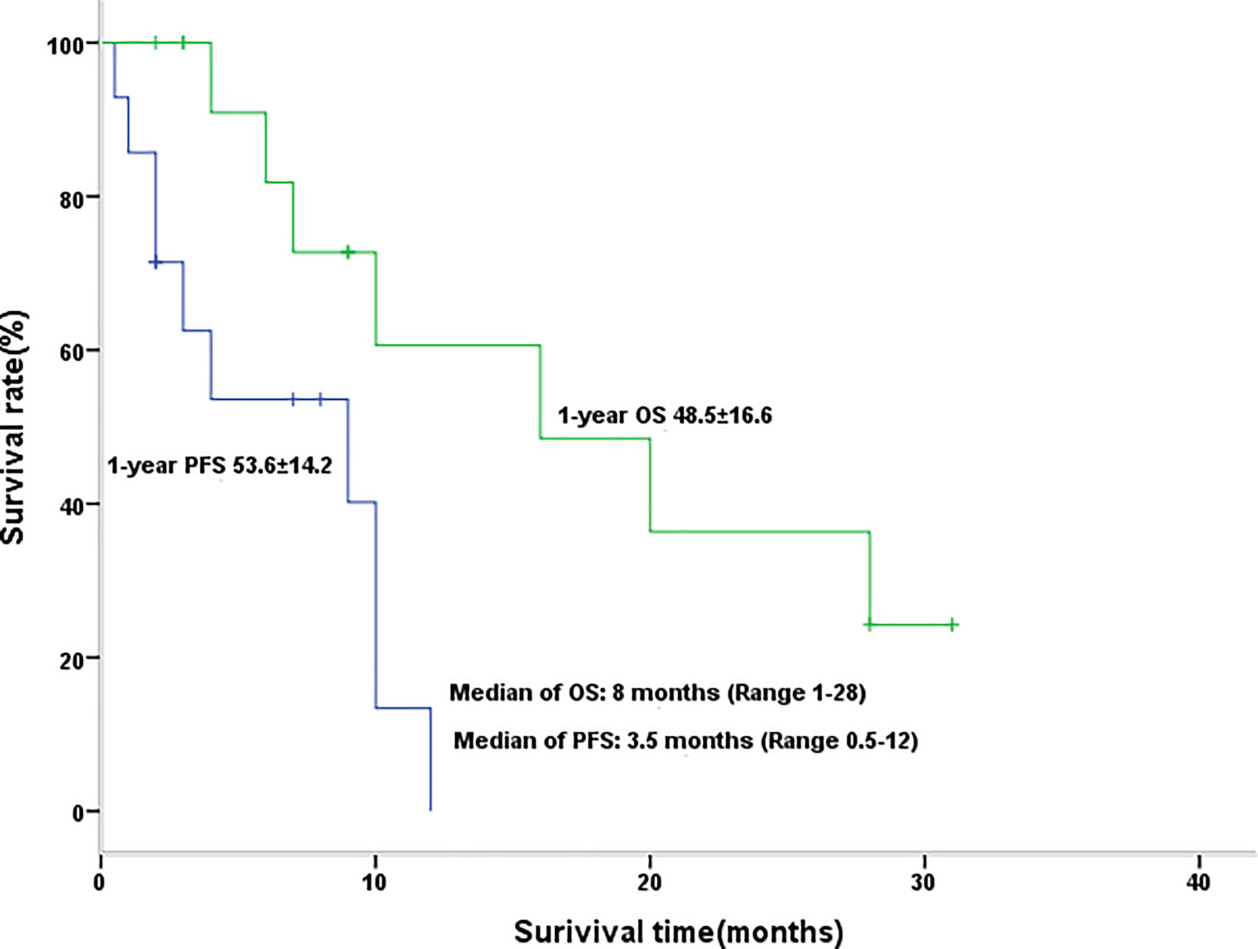
Kaplan-Meier graph for progression-free survival and overall survival in patients with efficacy assessment (n = 14).

The disease control rate (DCR) was 64% (of the 14 evaluable patients, two CR, five PR, and two SD), and the objective response rate (ORR) was 50% (two CR, five PR). The median PFS was 3.5 months (range 0.5–12 months), and the median survival duration was 8 months (range 1–28 months) (**Figure 1**, **Table 3**).

**Table 3:** Results and treatment of 16 cases of recurrent/refractory FH Wilms tumor.

No	Age^1^	Sex	Stage^2^	Chemotherapy before AI	No. of relapsed	Accumulation of THP (mg/m^2^)	Met sites	No. of AI	Response to AI	Surgery of met	RT ofMet sites	Follow-up(months)
1	4.2 y	F	II	VA, CAV/CE	2	150	Lung	2	PD	Yes, after AI	Yes, after AI	DOD, 3.5
2	7 y	M	IV	VAD, CAV/CE	2	350	Lung	2	SD	No	No	DOD, 10.9
3	3 y	M	III	VAD, CAV/CE, VIP	3	300	Lung	6	PR	Yes, after AI	Yes, after AI	NED, 10.1
4	2 y	F	III	VAD, CAV/CE	2	250	Liver, Abdominal cavity	3	SD	No	Yes, after AI	DOD, 16.6
5	8 y	M	II	VA, CAV/CE	2	250	Lung	4	PR	Yes, after AI	Yes, after AI	NED, 4.0
6	1 y	F	II	VA, CAV/CE	2	200	Celiac lymph nodes	6	CR	No	No	NED, 28.8
7	3 y	M	IV	VAD, CAV/CE	2	250	Lung	1	PD	Yes, after AI	Yes, after AI ^3^	NED, 3.7
8	4 y	M	III	VAD, CAV./CE, VIP	3	250	Pelvic mass	6	PR	No	Yes, after AI	AWD, 32.4
9	6 y	M	III	VA, CAV/CE	2	250	Liver, pelvic mass	3	PD	No	No	DOD, 4.3
10	10 y	M	II	CAVE, Act-D+CBP, VIP	2	400	Lung, Abdominal cavity	3	PD	No	No	DOD, 7.3
11	0.5 y	F	V	VAD, CAV/CE,VIP	2	350	Lung	2	PD	No	No	DOD, 28.6
12	5 y	F	IV	VAD, CAV/CE	2	300	Lung	8	CR	No	No	NED, 9.2
13	11 y	F	III	VAD, CAV/CE, VIP	2	200	Lung	6	PR	No	Yes, after AI	DOD, 20.9
14	4 y	F	IV	VAD, CyD/CE	Refractory	300	Lung	5	PR	No	Yes, after AI	NED, 2.6
15	4.2 y	M	IV	VAD, CAV/CE	Refractory^5^	300	Lung, bone	1	NA	NA	NA	AWD, 1.1
16	11 y	F	III	VA, CAV/CE, VIP	3	300	Abdominal cavity	6	NA	Yes, before AI	Yes, after AI	NED, 7.3

FH, favorable histology; AI, doxorubicin hydrochloride liposome, irinotecan; F, female; M, male; VA, vincristine, actinomycin; CAV, cyclophosphamide, pirarubicin, vincristine; CE, carboplatin, etoposide; VAD, vincristine, pirarubicin, actinomycin; VIP, etoposide, carboplatin, ifosfamide; CyD, cyclophosphamide, pirarubicin; Met, Metastasis; PD, progressive disease; SD, stable disease; PR, partial response; CR, complete response; NA, not available; DOD, dead of disease; NED, no evidence of disease; AWD, alive with disease; 1: age at relapse or refractory; 2: stage at the initial diagnosis; 3: Patient #7 did not receive whole-lung radiotherapy due to PD of the lung lesions after initial chemotherapy; 4: the bilateral lung metastases were refractory to initial treatment, and they were too many to operate; 5: the response of the right lung lesions was PD after first-line chemotherapy.

### Toxicity

A total of 16 patients were systemically assessed for toxicities (**Table 4**). No fatal adverse events or renal toxicity were observed, and modest adverse effects could be administered at the outpatient service (the patient received chemotherapy in the hospital department and was discharged after the completion of chemotherapy, and the toxicities of chemotherapy were treated in the outpatient department in our cancer center). The common grade 3 toxicity-related events were diarrhea (23%), abdominal pain (38%), and leukopenia (19%). The only grade 4 toxicity event was leukopenia (19%). Grade 1–2 vomiting and nausea were easily treated. Generally, grade 3 was manageable if antidiarrhea medications were routinely used. In this study, 11 patients received pegylated recombinant human granulocyte colony-stimulating factor (PEG-rhG-CSF) as an injection, and most patients had mild myelosuppression or febrile neutropenia. Mild cardiac (44%) and modest hepatic (13%) toxicity was observed. Several nonspecific symptoms, including mucositis and fatigue, occurred and were readily managed. None of the patients delayed chemotherapy because of the toxicity and side effects of chemotherapy.

**Table 4:** Possible treatment-related adverse events after treatment.

Toxicity	Grade 0	Grade I–II	Grade III	Grade IV
Non-hematological				
Diarrhea	1 (8%)	9 (69%)	3 (23%)	
Abdominal pain	0	8 (62%)	5 (38%)	
Vomiting	3 (21%)	10 (72%)	1 (7%)	
Nausea	1 (7%)	11 (79%)	2 (14%)	
Mucositis	5 (42%)	5 (42%)	2 (16%)	
Fatigue	2 (13%)	14 (87%)	0	
Alopecia	0	16 (100%)		
Alanine aminotransferase increased	15 (94%)	1 (6%)	0	
Aspartate aminotransferase increased	14 (87%)	2 (13%)	0	
Febrile neutropenia	11 (73%)	4 (27%)	0	
ECG performance status	9 (56%)	7 (44%)	0	
Hematological				
Leukopenia	3 (19%)	7 (43%)	3 (19%)	3 (19%)
Anemia	1 (7%)	13 (86%)	1 (7%)	
Thrombocytopenia	11 (74%)	2 (13%)	2 (13%)	

## Discussion

The prognosis of relapsed or refractory WT was poor, especially in high-risk relapsed patients who received three chemotherapy agents for frontline therapy. The AREN1921 study was a stage II prospective clinical trial (NCT04322318) investigating the treatment of newly diagnosed stage II–IV DA WTs or FH WTs that had returned (relapsed). According to the AREN1921 study, very high-risk relapsed FH WTs (those treated with three or more drugs for the initial WT) were treated with the regimen ifosfamide, carboplatin, etoposide (ICE)/cyclophosphamide/topotecan. However, in our study, all of the relapsed or refractory WT patients had received cyclophosphamide, etoposide, carboplatin, doxorubicin, and ifosfamide. In recent years, several retrospective studies have shown that irinotecan-containing regimens have a certain effect in recurrent WTs, but the combination of irinotecan and chemotherapy drugs is not uniform (21, 23–25). A International Society of Paediatric Oncology (SIOP) retrospective study showed that 14 patients with evaluable relapsed WT who received irinotecan-containing regimens (including vincristine, temozolomide, bevacizumab, etc.) had an ORR of 21.4%, and the response rate was not satisfactory (25). Anthracyclines were effective agents for patients with WT. Concerns about the cardiotoxicity of anthracyclines have restricted the dose of anthracyclines. Studies have revealed that doxorubicin-induced heart failure (HF) occurs in 3%–5% of patients treated with 400 mg/m^2^ doxorubicin (28). Cumulative doses of doxorubicin in patients with WT in COG and SIOP studies were no greater than 250 mg/m^2^ (22, 29). Doxorubicin hydrochloride liposomes are a novel formulation of doxorubicin encapsulated in polyethylene glycol-coated liposomes, and their pharmacokinetics (PK) is markedly different from that of doxorubicin. A study showed that patients exposed to relatively high cumulative doses, 540–840 mg/m^2^, did not show evidence of acute congestive HF, which suggests that doxorubicin hydrochloride liposomes might be less cardiotoxic than doxorubicin (30). Doxorubicin hydrochloride liposomes may become a potentially effective chemotherapeutic drug for children with relapsed and refractory WTs. Alternating doxorubicin hydrochloride liposomes with conventional anthracycline may improve the prognosis of relapsed or refractory WT patients. Irinotecan combined with doxorubicin hydrochloride liposomes may become effective rescue chemotherapy for relapsed and refractory WTs. Studies have shown that the maximum tolerated dose of doxorubicin hydrochloride liposomes administered every 4 weeks to pediatric patients is 60 mg/m^2^ (27). According to our experience in doxorubicin hydrochloride liposomes, in this study, we accepted the regimen of doxorubicin hydrochloride liposome as 40 mg/m^2^ for a single day of treatment.

In our study, among the 14 evaluable patients with FH WT, two patients achieved CR, five achieved PR after AI regimen chemotherapy, and the ORR was 50%, indicating that the AI regimen was effective for relapsed and refractory FH WTs. However, the SIOP study showed that irinotecan-containing regimens had poor efficacy in relapsed WT, with an ORR of 21.4% (25). The response rate of our study for patients with relapsed and refractory WTs is better than that of the SIOP clinical study, indicating that the efficacy of irinotecan combined with doxorubicin hydrochloride liposomes is superior to that of irinotecan-containing regimens (such as vincristine, temozolomide, and bevacizumab).

The COG AREN0321 clinical study showed that irinotecan combined with vincristine showed good efficacy in newly treated DA WT patients (22). The SIOP clinical study enrolled 14 patients with evaluable efficacy; eight patients experienced a first relapse, and nine patients had a high-risk histological type (tumors included those with diffuse anaplasia or blastemal-type histology after preoperative chemotherapy), including four DA WTs and five blastemal types (BTs). The ORRs of intermediate-risk histological type (tumors were stromal, epithelial, focal anaplasia, mixed, or regressive histology) and high-risk histological type WT patients were 33.3% and 11.1%, respectively. The results of the SIOP study indicate that the relapsed high-risk WT was not sensitive to irinotecan-containing salvage regimens. In the present study, all patients were diagnosed with FH WT. Whether the AI regimen was effective for high-risk histological type WT needs to be further explored.

Interestingly, of the 14 evaluable patients, one patient had local recurrence and achieved CR, the ORR of nine patients with isolated lung metastasis was 55.5% (one CR, four PR, one SD, three PD), and the ORR of four patients with both local and distant recurrence was 25% (one PR, one SD, two PD). These results indicate that the AI regimen may be more effective for solitary local or lung relapse patients. Nevertheless, sample size expansion is required to verify this conclusion.

In the present study, two CR patients achieved longer survival after AI regimen chemotherapy; four out of five PR patients who achieved CR after further clinical management (surgery or RT) survived at the last follow-up, and the response to the AI regimen was converted into a survival benefit; five PD patients had poor survival, most of whom died within 2 years, and a new therapeutic strategy needs to be explored.

In this study, the median number of treatment courses for patients receiving the AI regimen was 3 (1–8 courses), and the median cumulative dose of doxorubicin hydrochloride liposomes was 120 mg/m^2^ (40–240 mg/m^2^). Seven patients had mild abnormalities in the electrocardiogram, but none of the patients had severe cardiotoxicity (such as HF and arrhythmia). Because of the short follow-up time, a long time was needed to follow up on long-term cardiotoxicity. In the study, most patients relapsed more than two times and received high-intensity chemotherapy in the past. Considering that the AI regimen may cause severe bone marrow suppression, most patients were given long-acting granulocyte colony-stimulating factors to prevent neutropenia. The most common grade 3 side effects observed in this study included leukopenia (19%), abdominal pain (38%), diarrhea (23%), and mucositis (16%). The only grade 4 toxicity event was leukopenia (19%). The toxicity of the AI regimen was tolerable, with no treatment-related deaths, and none of the patients delayed treatment due to toxicity. Studies have shown that the dose-limiting toxicity of doxorubicin hydrochloride liposomes is mucositis (27). The incidence of mucositis in this study was not high, suggesting that doxorubicin hydrochloride liposomes at 40 mg/m^2^ are safe for relapsed and refractory WTs. Whether increasing the dose of doxorubicin hydrochloride liposomes can further improve the efficacy is worthy of exploration.

Of note, this is the first report of a therapeutic regimen combining these two agents. In this study, we noted that the adverse effects were commonly self-limiting and easily controllable with routine intervention, and this therapeutic regimen was generally continued without delayed therapy. Nevertheless, we acknowledge some limitations. As a retrospective single-arm study, the comparison could not be performed because this study lacks a control group, which may cause selection bias due to the non-randomized design. Furthermore, limited sample sizes were enrolled in this study. However, all patients with manageable adverse effects could continue the therapeutic regimen without delay of therapy, and this study provided valuable experience for the treatment of relapsed or refractory WT.

In conclusion, the combination regimen of irinotecan and doxorubicin hydrochloride liposomes indicates promising efficacy for relapsed or refractory WT patients with tolerable toxicities, especially for the FH WTs. A prospective clinical trial is warranted.

## Data Availability Statement

The original contributions presented in the study are included in the article/supplementary material. Further inquiries can be directed to the corresponding authors.

## Ethics Statement

The studies involving human participants were reviewed and approved by Sun Yat-sen University Cancer Center. Written informed consent to participate in this study was provided by the participants’ legal guardian/next of kin.

## Author Contributions

JW, LZ, LLG, YZZ, and XFS designed the study. LZ, YZ, SHZ and JW performed the analysis and drafted the manuscript. YQ, FFS, JZ, SYL, JTH, LHW, RQC, and ZJZ revised the manuscript. All authors contributed to the article and approved the submitted version.

## Conflict of Interest

The authors declare that the research was conducted in the absence of any commercial or financial relationships that could be construed as a potential conflict of interest.

## Publisher’s Note

All claims expressed in this article are solely those of the authors and do not necessarily represent those of their affiliated organizations, or those of the publisher, the editors and the reviewers. Any product that may be evaluated in this article, or claim that may be made by its manufacturer, is not guaranteed or endorsed by the publisher.
